# Case report: Adult patient with *WWOX* developmental and epileptic encephalopathy: 40 years of observation

**DOI:** 10.3389/fgene.2024.1477466

**Published:** 2024-10-23

**Authors:** Anna Teplyshova, Artem Sharkov

**Affiliations:** ^1^ Research Center of Neurology, Moscow, Russia; ^2^ Veltischev Research and Clinical Institute for Pediatrics and Pediatric Surgery of the Pirogov Russian National Research Medical University, Moscow, Russia; ^3^ Genomed Ltd, Moscow, Russia; ^4^ Shemyakin-Ovchinnikov Institute of Bioorganic Chemistry, Russian Academy of Sciences, Moscow, Russia

**Keywords:** developmental and epileptic encephalopathy, WWOX-DEE, epilepsy, WWOX, natural history

## Abstract

WWOX developmental and epileptic encephalopathy is characterised by drug-resistant epilepsy with onset within the first year of life and severe psychomotor developmental delay. This report presents for the first time a clinical case of an adult patient with a homozygous likely pathogenic variant (p.Thr12Met) in the *WWOX* gene, with more than 40 years of follow-up. The patient had refractory epilepsy with various types of seizures during his life: mainly epileptic spasms, autonomic, myoclonic, tonic seizures, and absences. The patient had a prominent developmental delay with a lack of expressive speech, but by the age of 3, he had acquired the skills to sit, crawl, and walk with support. In adolescence, there was an acute regression of acquired skills to a total absence of independent motor activity. The patient had dysmorphic features, such as upslanting palpebral fissures, arched eyebrows, and hypertelorism. For many years, the patient was given a diagnosis of cerebral palsy; 38 years after the onset of the disease, he was given a molecular genetic diagnosis of *WWOX*-associated developmental and epileptic encephalopathy. Our observation illustrates the natural history of *WWOX*-DEE and the high clinical significance of early genetic diagnostics for identifying the cause of developmental delay and resistant epilepsy.

## Introduction

Developmental and epileptic encephalopathies (DEEs) are a heterogeneous group of early-onset epilepsies associated with severe mental, motor, and speech impairments primarily caused by genetic factors. Recently, many genetic causes of DEEs have been discovered, primarily regulating ion transport, transcription translation, synaptic processes, cell growth and differentiation, as well as transport and metabolism of small molecules inside the cell and in the intercellular space.

The human gene *WWOX* (WW domain-containing oxidoreductase) is one of such monogenic causes of DEE that is located on chromosome 16q23.1-q23.2 and encodes the WWOX protein (46 kDa) consisting of 414 amino acids. This protein contains two N-terminal WW domains (referred to as WW1 and WW2) and a catalytic domain in the C-terminus, which is homologous to short-chain dehydrogenase/reductase (SDR) family proteins, although the substrate of WWOX is currently unknown. These domains enable WWOX to interact with numerous partner proteins, functioning as an adaptor protein and transcriptional repressor (Bednarek AK., et al., 2000).

Somatic mutations in the *WWOX* gene have been found in various neoplasms, highlighting its role as a tumour suppressor gene. Further genetic analysis using chromosomal microarray analysis or whole-exome sequencing identified biallelic pathogenic variants in *WWOX* in autosomal recessive neurological disorders: spinocerebellar ataxia type 12 (SCA12) accompanied by epilepsy and intellectual disability and childhood epileptic encephalopathy known as *WWOX*-related epileptic encephalopathy (WOREE) (Mallaret M., et al., 2014; Abdel-Salam G et al., 2014).

*WWOX*-developmental and epileptic encephalopathy (*WWOX*-DEE) is characterised by drug-resistant epilepsy with onset within the first year of life and severe psychomotor developmental delay. Microcephaly, retinal degeneration, and premature death between 2 and 4 years of age have been observed in severe cases (Mignot et al., 2015).

To date, approximately 700 pathogenic and likely pathogenic variants have been identified in the WWOX gene by ClinVar. However, approximately 98 variants are associated with epileptic encephalopathy. Notably, no specific region within the gene has been conclusively linked to WWOX-DEE.

The variant p. Thr12Met is located in the N-terminal domain and does not fall within any known domains. However, it is predicted as a tyrosine phosphorylation site by NetPhos 2.0 Server (Chiang MF et al., 2013).

Eighteen additional pathogenic or likely pathogenic variants have been reported in the N-terminal domain of patients with the DEE phenotype.

Previously, cases of DEE associated with the *WWOX* gene were more commonly described in children. We present a clinical case of an adult patient with a homozygous, likely pathogenic variant in the *WWOX* gene reported for the first time.

## Case report

The patient is a 40-year-old man, the second child of healthy, non-consanguineous parents. There was no family history of any neurodevelopmental disorders or epilepsy. Lack of foetal motor activity was recorded in the last month of pregnancy. The boy was born at 39–40 weeks of gestation, and the Apgar score was 7. He had mild asphyxia during childbirth that did not lead to disruption of the child’s activity in the first 4 months. His birth weight was 3,600 g, and his length was 53 cm.

### History of epilepsy

Prolonged (>30 min) febrile tonic–clonic seizures developed at the age of 4 months, and polymorphic unprovoked seizures occurred with high frequency thereafter. Several seizure types were seen: flexor epileptic spasms, which often occurred in clusters, autonomic seizures with impaired awareness, and myoclonic seizures. Epileptic spasms and autonomic seizures stopped by the age of 1.5 years. Between 1 and 12 years, tonic seizures with nods and falls (epileptic drop attacks) were observed. From childhood until now, the patient experienced myoclonic seizures, absences with eyelid myoclonia, and nonmotor unknown onset behaviour arrest seizures. Various antiseizure medications have been prescribed: phenobarbital, valproic acid, carbamazepine, topiramate, levetiracetam, ethosuximide, and perampanel. No sustained positive effect of treatment was detected. In recent years, the patient has been taking valproic acid (1,000 mg per day), levetiracetam (2000 mg per day), and perampanel (6 mg per day). The seizure frequency has remained extremely high throughout the disease course, reaching 10–30 paroxysms daily.

### Clinical features

Until 4 months of age, no significant developmental abnormalities were noted (normal muscle tone and sufficient amount of motor activity). The child babbled and could hold his head up by 1 month. The onset of epilepsy was followed by severe developmental (intellectual and motor) delay. The child could sit independently after 1 year, crawl from 1.5 years, and walk with support from 3 years of age.

Before 1 year, the verbal skills included pronunciation of separate sounds and syllables with no word production. After 1-year, rapid language regression started until verbalisation was completely lost. The child could react to addressed speech. He could show emotions consistent with the situation, track objects, and smile while listening to music.

After 12 years of life, a regression of acquired skills and a reduction of independent motor activity were noted; he ceased to walk and then sit on his own. At this time, scoliosis, kyphosis, and later upper and lower limb contractures formed.

Since infancy, the patient has often suffered from respiratory diseases and repeatedly experienced pneumonia. From 36 years of age, the frequency of pneumonia has increased to four times a year, often requiring inpatient treatment, and bulbar palsy has appeared, leading to tube feeding. After the age of 38 years, the patient’s respiratory failure deteriorated after contracting COVID-19, and ventilatory support was initiated, continuing until now.

**FIGURE 1:**
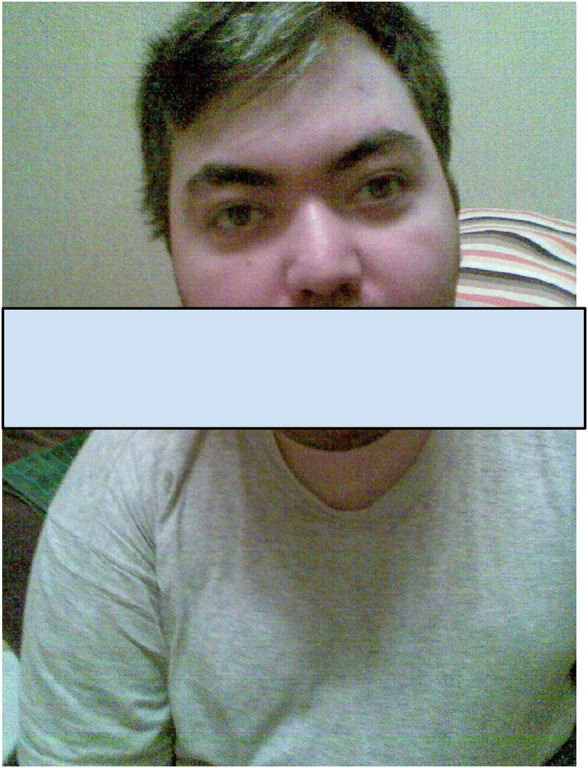
The photo of a patient at the age of 25 years showing dysmorphic features such as an upslanted palpebral fissure, highly arched eyebrows, and hypertelorism.

Currently, the patient’s height is 165 cm and weight is 60 kg. He is minimally responsive, can open his eyes, focus on objects, and react to addressed speech. He expresses some emotions, can smile when feeling well, and gets disappointed, offended, and cries. Speech is completely absent. Exodeviation of the left eye and dysphagia are present. He can hold his head up and sit with support. Severe tetraparesis, clonus in both feet, and bilateral Babinski sign are distinguished. Dysmorphic features, such as upslanted palpebral fissure, highly arched eyebrow, and hypertelorism, are observed (Figure 1).

**FIGURE 2:**
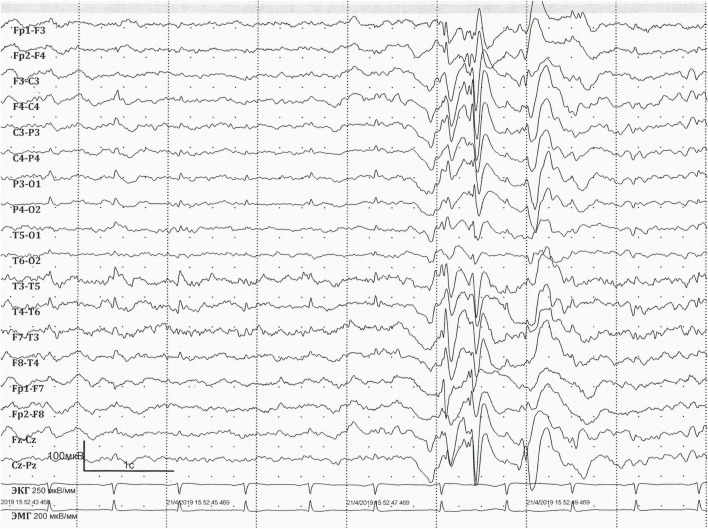
EEG at the age of 36 years. EEG longitudinal bipolar montage. Sweep: 30 mm/s; sensitivity: 100 mV/mm; bandpass: 1–70 Hz. Interictal EEG showing short generalised epileptiform discharges of irregular spike-, polyspike-and-slow-wave.

**FIGURE 3:**
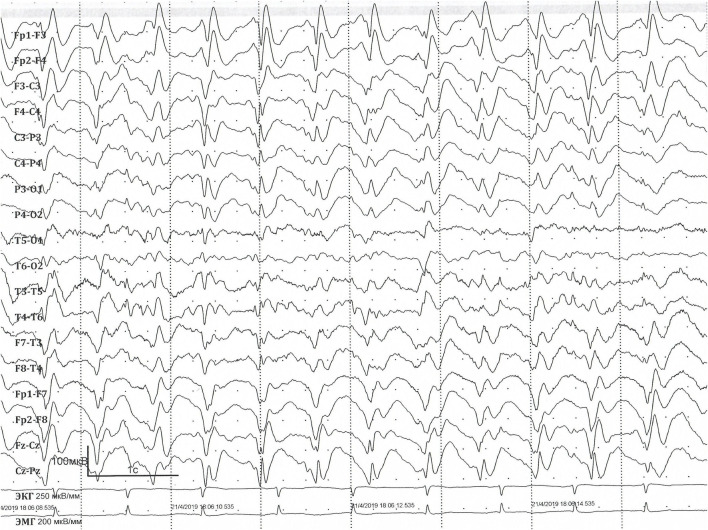
EEG at the age of 36 years. EEG longitudinal bipolar montage. Sweep: 30 mm/s; sensitivity: 100 mV/mm; bandpass: 1–70 Hz. Interictal EEG showing prolonged diffuse slow sharp-slow-wave complexes with the frequency of 1.5–2 Hz.

Long-term video EEG monitoring at the age of 36 years (Figures 2, 3) shows a background posterior dominant rhythm of 7–8 Hz. Sleep was divided into stages and phases; physiological patterns were well represented. Periodic regional slowing was registered in the fronto-centro-temporal areas, along with diffuse delta-slowing with amplitude predominance in the frontal regions. Short generalised epileptiform discharges of irregular spike-, polyspike-and-slow-wave complexes were recorded. In addition, epileptiform activity was represented by prolonged diffuse slow sharp-slow-wave complexes with a frequency of 1.5–2 Hz. The prevalence of epileptiform activity increased in sleep, reaching 70%–80% on some epochs, but overall remaining below 20%–25%. Ictal EEG showed epileptic generalised myoclonic seizures. Myoclonic seizures were registered in wakefulness, represented by shoulder jerks, eyelid myoclonia, and generalised myoclonic seizures, accompanied by generalised epileptiform discharges of irregular polyspike-slow-wave complexes on EEG.

Brain computed tomography at the age of 35 years did not show any significant pathological lesions. No magnetic resonance imaging of the brain was performed.

### Exome sequencing

Because the patient had febrile epileptic status, epileptic encephalopathy, and various types of seizures, a monogenic aetiology of epilepsy (primarily sodium channelopathies) was suspected, and whole-exome sequencing was carried out using a DNBSEQ-G400 and Agilent SureSelect Human All Exon V6 reagents for library preparation. Bioinformatic analysis was performed using an in-house software pipeline that included quality control of raw reads (FastQC tool v. 0.11.5), followed by read mapping to the hg19 human genome assembly (minimap2 v.2.24-r1122), sorting of the alignments, and marking duplicates (Picard Toolkit v. 2.18.14). Base recalibration and variant calling were performed with GATK3.8. Variant annotation was done using the Annovar tool (v.2018Apr16). The variant was leftover to hg38 by the UCSC LiftOver tool. CNV and SV analysis was performed using the Manta tool (v. 1.6.0). Further filtering was performed by functional consequences and population frequencies (less than 1% in The Genome Aggregation Database). The clinical significance of the variants was evaluated using ACMG criteria for variant interpretation (Richards et al., 2015). To validate the pathogenic variant in the proband and unaffected family members, Sanger sequencing was performed using the Applied Biosystems 3,130 xl Genetic Analyzer (HITACHI, Applied Biosystems Group of the Applera Corporation Japan, Waltham, MA, United States).

A homozygous variant in exon 1 of the *WWOX* gene (hg19, chr16:78133710C>T) NM_016373.4:c.35C>T was identified, leading to the replacement of an amino acid at position 12 (p.Thr12Met). This variant has not been described in the HGMD and ClinVar international databases of human mutations; however, there is a description of another amino acid substitution in the same position - p. (Thr12Arg), presented in the ClinVar database as a variant of unknown significance (RCV000690341.5) and as a pathogenic variant (RCV002286420.2).

The identified nucleotide sequence variant is registered in gnomAD ExomesVersion:4.1 in one heterozygous allele (ƒ = 0.000000702). The variant is a moderately conservative position (PhastCons100way 1.000, PhyloP100way 6.959), predominantly predicted by computer algorithms as possibly pathogenic (PrimateAI, SIFT, EIGEN—Pathogenic Supporting, MVP—Pathogenic Moderate, MutPred—Benign Moderate, and FATHMM, PROVEAN—Uncertain). The CADD score variant is 31. (Request: Chromosome 16, Position 78133710, CADD GRCh37-v1.7).

Given the full compliance with the clinical phenotype and according to the ACMG criteria, this variant of the nucleotide sequence is likely pathogenic (PM2, PM5, PP3, and PP4) and was uploaded in the ClinVar database at number RCV004689556.1.

Thus, the patient was given a molecular genetic diagnosis of *WWOX*-associated developmental and epileptic encephalopathy 38 years after the onset of the disease.

## Discussion

Approximately 80 patients with *WWOX*-DEE have been described in the literature, with the oldest being 23 years and 11 months old at date (Oliver et al., 2023).

For the first time, this article describes a 40-year-old adult patient with *WWOX*-DEE. Initial seizures in patients with *WWOX*-DEE typically occur in the first year of life, predominantly in the first few months (Oliver et al., 2023). The epilepsy onset in our patient occurred at 4 months of age as febrile epileptic status, consistent with previous reports of febrile seizures at disease onset (Piard J et al., 2019).

The clinical manifestations of epilepsy in patients with *WWOX*-DEE are variable and can include different types of seizures, both focal and generalised (Oliver et al., 2023). The frequency of epileptic seizures is very high (Piard J et al., 2019) and leads to epileptic encephalopathy. Among the epileptic syndromes observed in patients with *WWOX*-DEE, the most commonly reported cases include early infantile developmental encephalopathy, infantile spasms syndrome (previously known as West syndrome), and infantile epilepsy with migrating focal seizures (Battaglia L et al., 2023; Oliver et al., 2023; Piard Jet al., 2019; He J et al., 2020; Johannsen J et al., 2018; Mignot C et al., 2015; Shaukat Q et al., 2018). There are also observations where authors classify the syndrome as Lennox–Gastaut (Piard J et al., 2019; Johannsen J et al., 2018).

The presented patient more closely aligns with the criteria of Lennox–Gastaut syndrome, as evidenced by the presence of various seizure types, including tonic seizures, myoclonic seizures, and nonmotor seizures with behaviour arrest, with a specific EEG pattern showing generalised slow spike-and-wave complexes of <2.5 Hz. Although typical generalised paroxysmal fast activity (GPFA) in sleep was not detected, perhaps due to the lack of EEG at an early age, the patient can be diagnosed with Lennox–Gastaut syndrome (Specchio N et al., 2022).

In this case, we were able to track the dynamics of changes in seizure types over 40 years with *WWOX*-DEE. Similar observations have not been previously reported in the literature.

Epilepsy in patients with *WWOX*-DEE is typically resistant to antiseizure medications (Oliver et al., 2023). The therapy also did not yield a stable positive response in the presented patient.

The most commonly described prenatal anomalies in patients with *WWOX*-DEE included decreased foetal movement and macrosomia (Piard J et al., 2019). The presented patient exhibited a lack of motor activity in the later stages of the mother’s pregnancy. It is assumed that the *WWOX* pathogenic variants can lead to disorders in the development of the nervous system that manifest during embryonic development (Steinberg and Aqeilan, 2021).

All previously presented patients with *WWOX*-DEE experienced severe developmental delays and significant neurological symptoms progressing over time. The developmental delay in our case coincided with the onset of epilepsy. However, the patient acquired some motor skills during childhood. There was an acute regression in motor abilities, leading to severe tetraparesis from adolescence onwards. Speech development was impaired from the age of 1.

It is mentioned in the literature that patients with *WWOX*-DEE often experience respiratory disorders, including respiratory insufficiency, asthma, recurrent infections, and aspiration pneumonia (Khan MA et al., 2023). In the study by Piard et al. (2019), respiratory disorders were recorded in 40% of patients. Frequent respiratory illnesses occurred throughout our patient’s life from childhood and contributed to significant neurological regression in recent years.

Brain neuroimaging typically reveals various anomalies in patients with *WWOX*-DEE, such as disrupted myelination, decreased thickness of corpus callosum, white matter abnormalities, cerebral atrophy, and a characteristic pattern of severe frontotemporal atrophy, hippocampal atrophy, and optic nerve atrophy (Battaglia L et al., 2023; Oliver et al., 2023).

Patients with *WWOX*-DEE have a high mortality rate, estimated at approximately 35% (Oliver et al., 2023). The most commonly reported cause of death in these patients in the literature is respiratory complications (Havali C et al., 2021; He J et al., 2020; Mignot C et al., 2015; Weisz-Hubshman M et al., 2019). Other reported causes of mortality include status epilepticus (Abdel-Salam et al., 2014), obstructive cardiomyopathy (Valduga M et al., 2015), and sudden unexpected death in epilepsy (SUDEP) (Oliver et al., 2023). It has been shown that the risk of mortality correlates with the *WWOX* genotype, with the presence of at least one missense variant increasing the probability of 5-year survival from <50% to >75% (Oliver et al., 2023).

The phenotype of patients depends on the following combination: premature translation termination due to biallelic null (i.e., frameshift, nonsense, donor/acceptor, splice site, and deletion) variants lead to more severe phenotypes of early epileptic encephalopathy, while biallelic missense variants lead to a predominantly spinocerebellar ataxia phenotype. The phenotype of patients carrying null and missense variants falls in an intermediate range (Oliver et al., 2023).

Early identification of the genetic cause in this case could have influenced a better understanding of the disease prognosis and family expectations, as well as the tactics for disease prevention within the family. However, the impact on the choice of therapy at that time would have been negligible.

*WWOX* gene therapy is a сurrently promising therapy. Repudi S et al. (2021) designed and produced an adenoassociated viral vector (AAV9) harbouring murine Wwox or human *WWOX* cDNA and driven by the human neuronal Synapsin I promoter (AAV-SynI-WWOX). Testing the efficacy of AAV-SynI-WWOX delivery in Wwox-null mice demonstrated that specific neuronal restoration of *WWOX* expression rescued brain hyperexcitability and seizures, hypoglycemia, myelination deficits, and the premature lethality and behavioural deficits of Wwox-null mice. Further research in the field of gene therapy is needed.

## Conclusion

Our observation illustrates the high clinical significance of genetic diagnostics for identifying the cause of developmental delay and epilepsy, even in adult patients. Adult epileptologists should be wary of genetic pathology in drug-resistant epilepsy with onset in childhood. Knowledge of the pathophysiology underlying syndromes associated with the *WWOX* gene and its genotype–phenotype correlations is currently limited, hindering therapeutic possibilities. There is an urgent need to identify and classify all registered variants in *WWOX* to distinguish disease-causing alleles and their associated symptoms severity, as well as benign variants, to improve diagnostics and treatment.

## Data Availability

The data presented in the study are deposited in the ClinVar repository, accession number SCV005094612.1, First in ClinVar: Aug 11, 2024.
